# Laparoscopic hepatectomy for hepatocellular carcinoma in a patient with congenital factor V deficiency: a case report

**DOI:** 10.1186/s40792-022-01559-7

**Published:** 2022-10-22

**Authors:** Takaomi Seki, Mariko Tsukagoshi, Norifumi Harimoto, Kenichiro Araki, Akira Watanabe, Norihiro Ishii, Kei Hagiwara, Kouki Hoshino, Ryo Muranushi, Satoru Kakizaki, Yoshiyuki Ogawa, Hiroshi Handa, Ken Shirabe

**Affiliations:** 1 Department of General Surgical Science, Division of Hepatobiliary and Pancreatic Surgery, Graduate School of Medicine, Gunma University, 3-39-15, Showamachi, Maebashi, Gunma 371-8511 Japan; 2 Department of Clinical Research, National Hospital Organization Takasaki General Medical Center, 36 Takamatsucho, Takasaki, Gunma 370-0829 Japan; 3 Department of Hematology, Graduate School of Medicine, Gunma University, 3-39-15, Showamachi, Maebashi, Gunma 371-8511 Japan

**Keywords:** Factor V deficiency, Hepatocellular carcinoma, Laparoscopic hepatectomy

## Abstract

**Background:**

Factor V (FV) deficiency is an extremely rare disease, with an incidence of 1 in 1 million. The bleeding symptoms are mild, and the prognosis is good; however, the safety of surgical treatment is unclear, because there are few available reports. Herein, we report a case of hepatocellular carcinoma with congenital FV deficiency in a patient who safely underwent laparoscopic hepatectomy.

**Case presentation:**

A 79-year-old man, diagnosed with hepatocellular carcinoma of liver segment 5, with type C cirrhosis and sustained virological response visited our hospital. He had congenital FV deficiency, and blood tests showed coagulation deficiencies with an FV activity of < 2.6%, prothrombin time activity of 11%, and activated partial thromboplastin time of 100.3 s. Surgery and radiofrequency ablation were considered for treatment. Since the tumor was in contact with the Glissonean pedicle 5 + 6, surgery was judged to be superior from the viewpoint of safety and curability. After discussing the safety of the surgery with a hematologist, it was determined that the operation could be performed safely by transfusing sufficient fresh frozen plasma (FFP). Laparoscopic hepatic segment 5 + 6 subsegmental resection was performed with FFP transfusion, fluid restriction, airway pressure control, and central venous pressure reduction to control the bleeding. Bleeding was minimized during the transection of the liver parenchyma and no bleeding tendency was observed. The operative time was 445 min, and the amount of intraoperative bleeding was 171 mL. No complications, such as postoperative bleeding, were observed, and the patient was discharged on the eighth postoperative day.

**Conclusions:**

Liver surgery can be performed safely in FV-deficient patients with strict coagulation capacity monitoring and appropriate transfusion of FFP. Preoperative evaluation of cardiac function to determine tolerance to high doses of FFP and ingenuity of surgery and intraoperative management to minimize blood loss are important.

## Background

Factor V (FV) deficiency is an extremely rare disease with an incidence of 1 in 1 million [1]. Clinical symptoms include prolonged prothrombin time (PT) and activated partial thromboplastin time (APTT), resulting in skin and mucosal bleeding, menorrhagia, and intramuscular bleeding. The bleeding symptoms are mild, and the prognosis is good; however, the safety of surgical treatment is unclear, because there are few available reports. To the best of our knowledge, there have been no reports of hepatectomy in patients with FV deficiencies. Herein, we report a case of hepatocellular carcinoma (HCC) with congenital FV deficiency in a patient who underwent laparoscopic hepatectomy safely.

## Case presentation

**Fig. 1:**
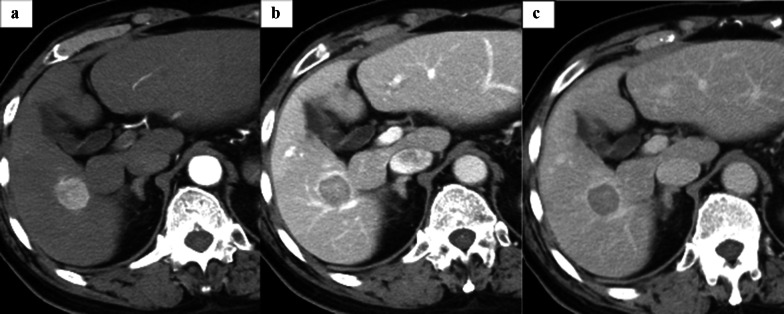
Dynamic abdominal CT imaging findings. **a** Arterial phase, **b** portal vein phase, **c** delayed phase. A nodule measuring 24 mm was found in hepatic segment 5, which was deeply stained in the arterial phase and washed out during the delayed phase

**Fig. 2:**
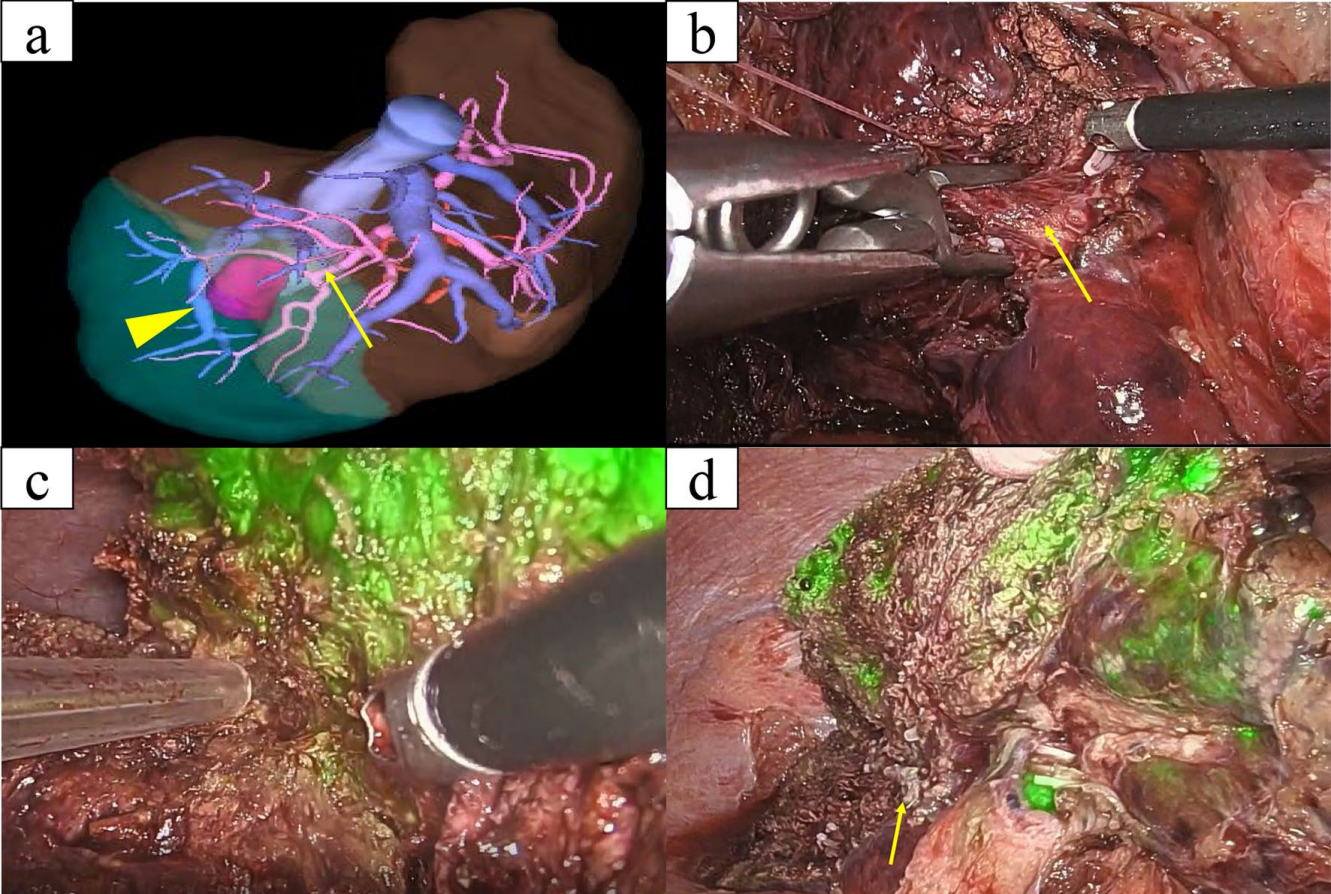
Intraoperative findings. **a** Three-dimensional image of hepatectomy simulation. The tumor was found in the hepatic segment 5 of the cranial side of the right hepatic vein (arrowhead). We performed a simulation in which P5 + 6 (arrow) was scheduled to be resected. **b** Captured image after clamping the Glissonean pedicle 5 + 6 (arrow). **c** Cut surface of the liver during transection of the liver parenchyma. **d** Cut surface of the liver after hepatectomy. The Glissonean pedicle 5 + 6 (arrow) is collectively resected using the Signia™ stapling system (Covidien Japan, Tokyo)

**Fig. 3:**
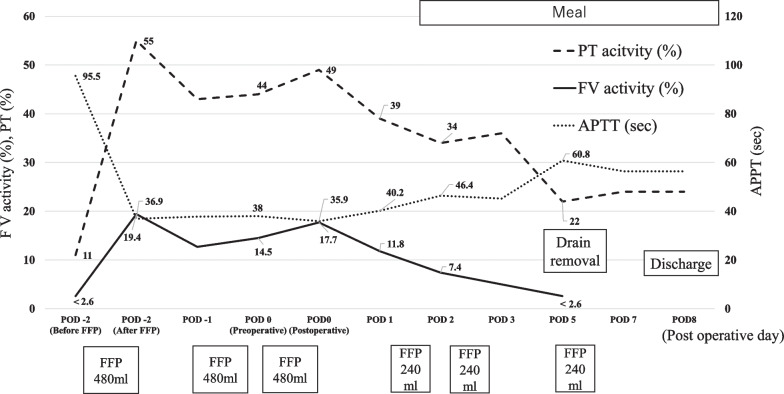
Perioperative treatment course and changes in coagulation data. FV activity increased with FFP transfusion and remained at approximately 15%. The patient consumed food on the first night after the operation, the drain was removed on the fifth day postoperatively, and the patient was discharged on the eighth day postoperatively. *FV* factor V, *PT* prothrombin time, *APTT* activated partial thromboplastin time, *FFP* fresh frozen plasma, *POD* postoperative day

**Fig. 4:**
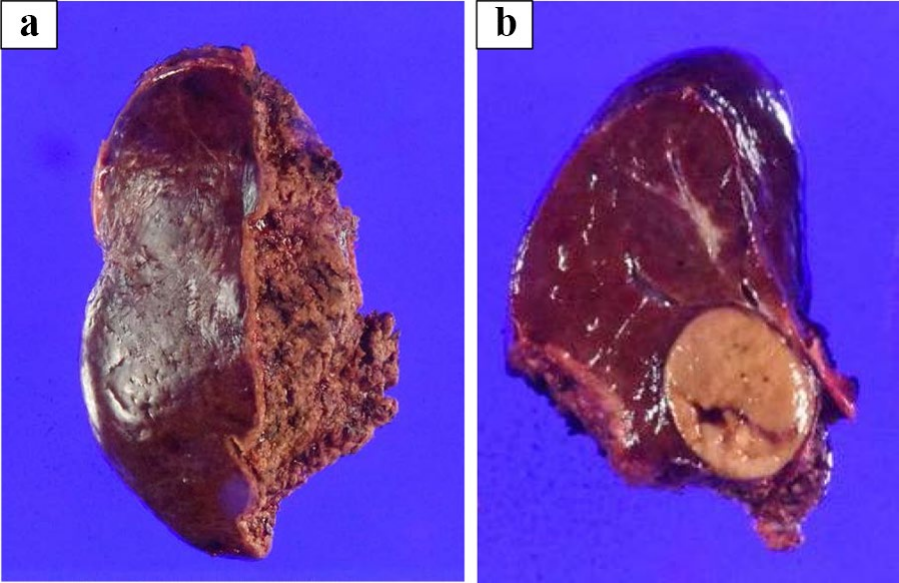
Macroscopic findings. **a** Resection specimen of the hepatic segment 5 + 6 subsegmental. **b** Cross section of the tumor. The tumor was 2.6 × 2.1 × 1.8 cm in size with a capsule and a well-defined white nodule

A 79-year-old man with a sustained virological response due to liver cirrhosis secondary to hepatitis C virus infection was diagnosed with a liver tumor on regular abdominal ultrasonography. The tumor, suspected to be HCC, was found in liver segment 5 on enhanced computed tomography (CT), and the patient was referred to our department for detailed examination and treatment. The patient was diagnosed with congenital FV deficiency approximately 20 years ago, and was hospitalized twice for epistaxis. As indicated in Table 1, blood tests showed markedly decreased FV activity of < 2.6%, PT activity of 11%, and prolonged APTT by 100.3 s, which are indicative of coagulopathy. Other coagulation factor activities, were within the normal range. The patient’s FV activity had been tested several times over the past 20 years and consistently resulted in markedly decreased FV activity. No FV inhibitors were detected using the Bethesda assay at our hospital [2, 3]. Cross-mixing tests revealed a pattern of coagulation factor deficiency. Although he had not undergone genetic testing, based on the blood test results and the reproducibility and ubiquity of these blood tests, he was diagnosed with congenital FV deficiency. The liver function reserve was Child–Pugh grade B (7 points). Liver damage was grade A, and the hepatic reserve was judged to be good [4]. As no esophageal varices, gastric varices, or splenomegaly were observed, severe portal hypertension was ruled out. We consulted a cardiologist regarding the patient's cardiac function. The patient’s exercise tolerance was good, and no abnormalities were observed in active cardiac conditions and the revised cardiac risk index [5, 6]. Based on the above findings, the patient's cardiac function was considered acceptable for surgery, although caution was advised against excessive fluid infusion. Pulmonary function was good. Computed tomography (CT) showed a 24 mm-sized nodule in liver segment 5, which was deeply stained in the arterial phase and washed out in the delayed phase (Fig. 1). Magnetic resonance imaging also showed a contrast effect in the segment 5 tumor in the early phase and wash-out in the delayed phase. A well-differentiated HCC was suspected. Based on the radiological findings, the patient was diagnosed with HCC (T2N0M0 stage II, according to the 8th edition of the Union for International Cancer Control staging system). Based on the Barcelona Clinic Liver Cancer (BCLC) staging algorithm, this case was regarded as early stage (A). It was confirmed that surgery was recommended as the tumor was an isolated nodule, and portal pressure and bilirubin were normal. We also considered radiofrequency ablation (RFA); however, since the tumor was in contact with the Glissonean pedicle 5 + 6 and RFA may cause bile duct injury due to a rapid increase in intratumoral pressure during cauterization, surgery was judged to be superior from the viewpoint of safety and curability. However, invasive treatment involved a high risk due to the coexistence of congenital FV deficiency. After discussing the safety of the operation with a hematologist, we decided that surgery was possible by transfusing fresh frozen plasma (FFP) during the perioperative period, maintaining FV activity at ≥ 15% and closely monitoring PT and APTT. Two days before the operation, 480 mL (approximately 7.4 mL/kg) of FFP was transfused, coagulation FV activity was ≥ 15% was confirmed, and PT and APTT were reduced. As our hospital can only measure FV activity once per week, we estimated the necessary FFP based on the above results and created an FFP transfusion plan. The FV activity immediately before the operation was 14.5%, and the operation was performed with a transfusion of 480 mL of FFP. The changes in perioperative coagulation data are presented in Table 2. The surgical procedure and results of this case were as follows: the patient was placed in the left lateral decubitus position, and six ports, including a Pringle method taping port, were used. The pneumoperitoneum pressure was maintained at 10 mmHg. An ultrasonic scalpel and Cavitron Ultrasonic Surgical Aspirator (CUSA; AMCO Inc., Tokyo, Japan) were used for the incision of the liver parenchyma. The tip of the CUSA was connected to a VIO 300 D generator (ERBE Elektromedizin GmbH, Tubingen, Germany), and hemostasis was performed in soft coagulation mode using bipolar forceps held in the operator's left hand. Intermittent pedicle clamps (15-minocclusion, 5-min reperfusion, Pringle method) were used during parenchymal cutting. Hemodynamic control aimed at maintaining low central venous pressure (< 5 mmHg) under anesthesia and weight loss perfusion minimizes blood loss Airway pressure was maintained at 14–16 cmH_2_O, reducing central venous pressure and volumetric perfusion. We adjusted the volume of transfusion during liver parenchymal cutting to approximately 5 mL/kg/h and aimed to achieve a urine volume of 0.5 mL/kg/h. Preoperative three-dimensional simulation was conducted using a SYNAPSE VINCENT volume analyzer (Fujiflim Co., Tokyo, Japan) (Fig. 2a). To resect the Glissonean pedicle 5 + 6, we first secured it by clamping (Fig. 2b). Segments 5 and 6 of the liver were identified by negative staining using indocyanine green fluorescent imaging navigation, and hepatectomy was performed Bleeding was minimized during the transection of the liver parenchyma and no bleeding tendency was observed (Fig. 2c). After hepatectomy, no bleeding was observed on the cut surface of the liver (Fig. 2d). Tachosil^®^ was applied around the hepatic vein and Gleason stump, and Bolheal^®^ was dripped onto the cut surface of the liver. Surgical procedures included laparoscopic hepatic segment 5 + 6 subsegmental resection and cholecystectomy. The operative time was 445 min, and the amount of intraoperative bleeding was 171 mL. The cumulative time of the Pringle method was 208 min and 51 s. Immediately after the operation, FV activity was 17.7%, and no bleeding from the wound or drain was observed. FFP (240 mL) was transfused on postoperative days 1 and 2. FV activity decreased to < 2.6% on the fifth postoperative day; therefore, another 240 mL of FFP was transfused and the drain was removed. The postoperative course was uneventful, and the patient was discharged 8 days after the operation (Fig. 3). Postoperative pathological examination revealed that the tumor was macroscopically demarcated and accompanied by a capsule, which was diagnosed as a stage IB (pT1bN0M0) HCC (Fig. 4). One month after the surgery, no complications were observed.

**Table1:** Preoperative blood test results

Inspection items	Results	Reference range
WBC (10^3^/μl)	6.9	4.0–9.6
Hb (g/dl)	14.9	13.2–17.3
Platelet count (10^3^/μl)	179	160–350
T-bil (mg/dL)	0.4	0.3–1.2
D-Bil (mg/dL)	0.04	0.0–0.2
AST (U/L)	30	13–33
ALT (U/L)	34	8–42
Alb (g/dl)	4.4	3.9–5.0
BNP (pg/mL)	5.6	0–18.4
ICG-R15 (%)	10.9	
PT activity (%)	11	0.85–1.23
PT-INR	4.28	
APTT (second)	100.3	27.0–39.0
Factor I (%)	82.9	70–120
Factor V (%)	2.6	70–120
Factor VII (%)	98.3	70–120
Factor VIII (%)	183.7	70–120
Factor IX (%)	108.3	70–120
Factor X (%)	85	70–120
Factor XI (%)	101.2	70–120
Factor XII (%)	86.3	70–120
Von Willebrand factor (%)	183	70–120
Factor V inhibitor	(–)	
Factor VIII-like antigen (%)	269	70–120

*PT* prothrombin time, *APTT* activated partial thromboplastin time

**Table 2:** Perioperative blood coagulation data transition

	POD-2 (Before FFP)	POD -2 (After FFP)	POD-1	POD 0 (Preoperative)	POD0 (Postoperative)	POD 1	POD 2	POD 3	POD 5	POD 7
PT activity (%)	11	55	43	44	49	39	34	36	22	24
PT-INR	4.02	1.31	1.48	1.46	1.39	1.57	1.64	2.31	2.31	2.08
APTT (s)	95.5	36.9	37.8	38	35.9	40.2	46.4	45.2	60.8	56.4
Factor V activity (%)	< 2.6	19.4	12.7	14.5	17.7	11.8	7.4	–	< 2.6	–

*POD* postoperative day, *FFP* fresh frozen plasma, *FV* factor V, *PT* prothrombin time, *APTT* activated partial thromboplastin time

## Discussion

FV is a coagulation factor that acts in the blood coagulation cascade; it is synthesized mainly in the liver, and concentration may decrease when hepatic synthesis function declines. Although most FV are present in the plasma, approximately 20% of circulating FV are found within platelet α-granules [7]. FV deficiency is a coagulopathy caused by quantitative deficiency and dysfunction of FV, and is a rare disease [1]. FV deficiency is classified as congenital or acquired depending on the presence or absence of an FV inhibitor. Congenital FV deficiency is caused by mutations in the FV gene itself, or by mutations in genes that affect FV processing and storage. In contrast, acquired FV deficiency has been reported to be triggered by malignant tumors, blood transfusions, collagen diseases, antibiotic use, and infectious diseases, such as HIV and tuberculosis. This patient was diagnosed with congenital FV deficiency approximately 20 years prior to presentation. At the first visit, we considered the possibility of acquired FV deficiency due to HCC or another coagulopathy. Although we did not perform genetic testing, we diagnosed congenital FV deficiency by conducting a detailed medical interview, confirming the clinical course, measuring the coagulation factor set, confirming the FV inhibitor by Bethesda assay and performing a cross-mixing test [2, 3]. Dissimilar to patients with hemophilia A and B, patients with FV deficiencies have prolonged PT and APTT, resulting in cutaneous and mucosal bleeding, menorrhagia, and intramuscular bleeding. There is no FV preparation for the treatment of bleeding; hence, FV was supplemented with FFP transfusion. Upon FFP transfusion, symptoms are milder and more asymptomatic than those in case of hemophilia; the risk of death associated with bleeding events is low, and the prognosis is good [7]. There are multiple reports of FFP transfusion for surgeries, such as heart surgery [8], cochlear implant surgery [9], intracranial drainage for chronic subdural hematoma [10], laparoscopic-assisted vaginal hysterectomy, bilateral salpingo-oophorectomy [11], and laparoscopic cholecystectomy [12]. In this case, surgery or RFA was considered for treatment based on the BCLC staging algorithm [13]. However, RFA posed a risk as the tumor was in contact with the Glissonean pedicle 5 + 6 and could potentially cause biloma formation [14]. In addition, due to the cooling effect of blood flow near the tumor, the temperature of the tumor may not rise sufficiently; hence, insufficient coagulation necrosis was a possibility [15]. As for the surgery, the hepatic reserve was classified as Child–Pugh grade B owing to decreased PT activity. It was, however, possible that hepatic reserve was not correctly evaluated due to the effect of FV deficiency. Liver damage was assessed as grade A using another hepatic reserve evaluation index [4], and the liver reserve was judged as good. The functional remnant liver volumetry criteria stipulated by our department were cleared [16]; thus, it was possible to perform surgery. The choice of surgery was further supported when cardiologists detected no abnormalities during cardiac function evaluation, and the hematologist advised sufficient hemostasis could be expected during surgery. To the best of our knowledge, this is the first report of hepatectomy performed in a patient with FV deficiency. Highly invasive surgery can be safely performed with appropriate FFP replacement therapy; hence, it is desirable to establish an appropriate protocol.

It is important to determine the preferred surgical procedure and its ingenuity to reduce the risk of bleeding. Laparoscopic surgery has been reported to minimize bleeding compared with open surgery [17]and, hence, is preferable. Hepatic blood flow was controlled by the Pringle method, indocyanine green fluorescent imaging navigation was used to identify the S5 + 6 subsegment of the liver, and CUSA was used as the main device for the transection of the liver parenchyma [18–21]. During intraoperative management, limiting fluid replacement, controlling airway pressure, and reducing central venous pressure and volumetric perfusion helped to reduce bleeding [22–25]. Cooperation within the perioperative team, including anesthesiologists, is essential to minimize bleeding.

In liver surgery for patients with FV deficiency, preoperative cardiac function should be evaluated to determine whether a large FFP dose can be tolerated. As mentioned above, cardiologists assessed cardiac function in our patient, which is generally performed by BNP, echocardiography, active cardiac conditions [5], and the revised cardiac risk index [6].Although it is necessary to supplement coagulation factors to improve coagulation function, FFP is transfused, as there are no FV-specific concentrated preparations. It is recommended that FV activity be maintained above 15–20% for surgery. A recommended protocol for FFP transfusion to increase FV activity, is: one dose of 15 to 25 mL/kg before surgery, 10 mL/kg every 12 h as needed, and an additional 10 mL/kg every 12 h as needed [26]. Overall, 2160 mL of FFP was transfused perioperatively. If FFP does not sufficiently improve coagulation function or FFP overdose is difficult to manage, platelet transfusion can be performed [26]. This is possible as approximately 20% of FV is present in platelet α-granules, and FV is released with the activation of platelets, resulting in a local FV concentration at least 100 times more than that of plasma. This approach can prevent FFP overloading and may be useful in some cases [27, 28]. In particular, FFP transfusion is less effective, and platelet transfusion is more useful in patients with acquired FV deficiency who have FV inhibitor [28, 29]. In cases of acquired autoimmune pathologies, free anti-FV autoantibodies are present in circulating plasma, causing antigen–antibody reactions with FV in the infused FFP, resulting in the loss of coenzyme activity of FV, and it is thought that the appropriate hemostatic effect may not be obtained. Platelet transfusion is effective because, as mentioned above, FV stored in platelet α granules is released at the bleeding site with platelet activation and works efficiently at the bleeding site.

In this case, FV activity was used as an index to monitor coagulation, because FV activity can be rapidly measured at our hospital once per week. As shown in Table 2, PT-INR, PT, and APTT were correlated with FV activity and were useful for evaluating coagulability. If FV activity cannot be measured, clinical symptoms, such as bleeding, and monitoring of PT-INR, PT and APTT, may be sufficient. In this case, PT activity of approximately 50%, PT-INR of approximately 1.5%, and APTT of about 40 s corresponded to an FV activity of 15%. However, the extent to which FFP transfusion improves PT-INR, PT, and APTT may vary depending on the individual constitution and institution that performs the measurement. It is important to transfuse FFP preoperatively and evaluate the degree of correlation between PT-INR, PT, and APTT.

## Conclusions

For the first time, we reported a case of hepatectomy for a patient with HCC and congenital FV deficiency. Liver surgery in this case was performed safely by evaluating cardiac function for operative tolerance in anticipation of high doses of FFP, strict perioperative monitoring of coagulation, evaluating the ingenuity of surgery, and performing intraoperative management to minimize blood loss.

## Data Availability

All data generated or analyzed during this study are included in the published article.

## Abbreviations

FV: Factor V
HCC: Hepatocellular carcinoma
CT: Computed tomography
PT: Prothrombin time
APTT: Activated partial thromboplastin time
FDG: 18F-fluorodeoxyglucose
BCLC: Barcelona clinic liver cancer
RFA: Radiofrequency ablation
FFP: Fresh frozen plasma

## References

[1] Mannucci PM, Duga S, Peyvandi F (2004). Recessively inherited coagulation disorders. Blood.

[2] Kasper CK, Aledort L, Aronson D, Counts R, Edson JR, van Eys J (1975). Proceedings: a more uniform measurement of factor VIII inhibitors. Thromb Diath Haemorrh.

[3] Verbruggen B, Novakova I, Wessels H, Boezeman J, van den Berg M, Mauser-Bunschoten E (1995). The Nijmegen modification of the Bethesda assay for factor VIII: C inhibitors: improved specificity and reliability. Thromb Haemost.

[4] Ikai I, Arii S, Kojiro M, Ichida T, Makuuchi M, Matsuyama Y (2004). Reevaluation of prognostic factors for survival after liver resection in patients with hepatocellular carcinoma in a Japanese nationwide survey. Cancer.

[5] Fleisher LA, Beckman JA, Brown KA, Calkins H, Chaikof EL, Fleischmann KE (2007). ACC/AHA 2007 Guidelines on Perioperative Cardiovascular Evaluation and Care for Noncardiac Surgery: Executive Summary: a Report of the American College of Cardiology/American Heart Association Task Force on Practice Guidelines (Writing Committee to Revise the 2002 Guidelines on Perioperative Cardiovascular Evaluation for Noncardiac Surgery) Developed in Collaboration With the American Society of Echocardiography, American Society of Nuclear Cardiology, Heart Rhythm Society, Society of Cardiovascular Anesthesiologists, Society for Cardiovascular Angiography and Interventions, Society for Vascular Medicine and Biology, and Society for Vascular Surgery. J Am Coll Cardiol.

[6] Lee TH, Marcantonio ER, Mangione CM, Thomas EJ, Polanczyk CA (1999). Derivation and prospective validation of a simple index for prediction of cardiac risk of major noncardiac surgery. Circulation.

[7] Huang JN, Koerper MA (2008). Factor V deficiency: a concise review. Haemophilia.

[8] Yotsumoto G, Masuda H, Toyokawa K, Iguro Y, Kinjo T, Sakata R (2005). Off-pump coronary artery bypass grafting in a patient with congenital factor V deficiency: report of a case. Surg Today.

[9] Fehdi MA, Lazraq M, Benhamza S, Bensaid A, Miloudi Y, Harrar NE (2020). Congenital factor V deficiency: perioperative management (case report). Pan Afr Med J.

[10] Meidert AS, Kinzinger J, Möhnle P, Pekrul I, Spiekermann K, Thorsteinsdottir J (2019). Perioperative management of a patient with severe factor V deficiency presenting with chronic subdural hematoma: a clinical report. World Neurosurg.

[11] Koduri PR, Kamineni V, Vedantham H, Joshi N (2016). Laparoscopic surgery in a woman with factor V deficiency: revisiting platelet factor V. Haemophilia.

[12] Zhang YH, Hong DF, Hu ZM, Wu WD, Zhang CW (2015). Successful laparoscopic common bile duct exploration in a patient with factor V deficiency, a case report and review of literature. Int J Clin Exp Med.

[13] Reig M, Forner A, Rimola J, Ferrer-Fàbrega J, Burrel M, Garcia-Criado Á (2022). BCLC strategy for prognosis prediction and treatment recommendation: the 2022 update. J Hepatol.

[14] Chang IS, Rhim H, Kim SH, Kim YS, Choi D, Park Y (2010). Biloma formation after radiofrequency ablation of hepatocellular carcinoma: incidence, imaging features, and clinical significance. AJR Am J Roentgenol.

[15] Yamakado K, Nakatsuka A, Ohmori S, Shiraki K, Nakano T, Ikoma J (2002). Radiofrequency ablation combined with chemoembolization in hepatocellular carcinoma: treatment response based on tumor size and morphology. J Vasc Interv Radiol.

[16] Araki K, Harimoto N, Kubo N, Watanabe A, Igarashi T, Tsukagoshi M (2020). Functional remnant liver volumetry using Gd-EOB-DTPA-enhanced magnetic resonance imaging (MRI) predicts post-hepatectomy liver failure in resection of more than one segment. HPB (Oxford).

[17] Wakabayashi G, Cherqui D, Geller DA, Buell JF, Kaneko H, Han HS (2015). Recommendations for laparoscopic liver resection: a report from the second international consensus conference held in Morioka. Ann Surg.

[18] Araki K, Harimoto N, Ishii N, Tsukagoshi M, Igarashi T, Watanabe A (2020). Optimal indications for an intercostal port for the superior segments in laparoscopic partial liver resection. Asian J Endosc Surg.

[19] Tsukagoshi M, Harimoto N, Araki K, Kubo N, Watanabe A, Igarashi T (2020). Liver metastasis from papillary thyroid carcinoma treated by laparoscopic hepatectomy 43 years after resection of the primary tumor: a case report. Surg Case Rep.

[20] Nomi T, Hokuto D, Yoshikawa T, Matsuo Y, Sho M (2018). A novel navigation for laparoscopic anatomic liver resection using indocyanine green fluorescence. Ann Surg Oncol.

[21] Ishizawa T, Saiura A, Kokudo N (2016). Clinical application of indocyanine green-fluorescence imaging during hepatectomy. Hepatobiliary Surg Nutr..

[22] Jones RM, Moulton CE, Hardy KJ (1998). Central venous pressure and its effect on blood loss during liver resection. Br J Surg.

[23] Iguchi T, Ikegami T, Fujiyoshi T, Yoshizumi T, Shirabe K, Maehara Y (2017). Low positive airway pressure without positive end-expiratory pressure decreases blood loss during hepatectomy in living liver donors. Dig Surg.

[24] Kobayashi S, Honda G, Kurata M, Tadano S, Sakamoto K, Okuda Y (2016). An experimental study on the relationship among airway pressure, pneumoperitoneum pressure, and central venous pressure in pure laparoscopic hepatectomy. Ann Surg.

[25] Egger ME, Gottumukkala V, Wilks JA, Soliz J, Ilmer M, Vauthey JN (2017). Anesthetic and operative considerations for laparoscopic liver resection. Surgery.

[26] Peyvandi F, Menegatti M (2016). Treatment of rare factor deficiencies in 2016. Hematology Am Soc Hematol Educ Program.

[27] Drzymalski DM, Elsayes AH, Ward KR, House M, Manica VS (2019). Platelet transfusion as treatment for factor V deficiency in the parturient: a case report. Transfusion.

[28] Chediak J, Ashenhurst JB, Garlick I, Desser RK (1980). Successful management of bleeding in a patient with factor V inhibitor by platelet transfusions. Blood.

[29] Franchini M, Lippi G (2011). Acquired factor V inhibitors: a systematic review. J Thromb Thrombolysis.

